# Postoperative Complications in Genioplasty and Their Association with Age, Gender, and Type of Genioplasty

**DOI:** 10.1155/2021/8134680

**Published:** 2021-11-17

**Authors:** Muslim Khan, Nigam Sattar, Mahwish Erkin

**Affiliations:** ^1^Khyber College of Dentistry, Peshawar, Pakistan; ^2^Islamic International Dental College, Islamabad, Pakistan; ^3^Shifa College of Dentistry, Islamabad, Pakistan

## Abstract

**Objective:**

The objective of our study was to determine the incidence of postoperative complications associated with different kinds of genioplasties done with or without concomitant orthognathic surgeries.

**Materials and Methods:**

Patients in whom facial asymmetry was corrected by genioplasty with age ranging from 16 to 55 at the Department of Oral and Maxillofacial Surgery were included in the study. Patients with facial asymmetry due to congenital problems were excluded.

**Results:**

59 patients were included, of which 38 were males and 21 were females with the age range of 16–55 (mean: 27.3729, Std. deviation: 4.70472). Advancement genioplasty was performed in 15.3%, reduction genioplasty was done in 16.9%, and advancement genioplasty with rotation was performed in 67.8% cases. In 28.8% cases, genioplasty was performed as an adjunct procedure with orthognathic surgery, and in 71.2% cases, genioplasty was performed alone. 3.4% patients developed infection, 8.5% had hematoma, and 6.8% had temporary numbness. Postoperative complications were seen more in men than in women. Correction of chin asymmetry by rotation/advancement showed the highest number of complications (84%).

**Conclusion:**

Temporary neurosensory disturbance of the inferior alveolar nerve is the most common complication that occurs after genioplasty. Patients shall be counselled preoperatively, and informed consent shall be obtained prior to surgery.

## 1. Introduction

Facial aesthetics play an important role in the personality and beauty of a person [1]. One of the most dominating features in the face is chin projection and shape. It is regarded as a significant characteristic of facial attractiveness [2]. Symmetry and harmonious proportion of the chin to the upper and middle third of the face are very important. Significant deformity in the chin can give an unaesthetic look in an otherwise aesthetically pleasant facial profile. Chin deformities can be defined as (a) excessive chin, (b) deficient chin, (c) asymmetrical chin, or a combination of these [3]. Surgical alteration of the chin has been used for many years to achieve proportional lower third of the face to the upper and middle third of the face [4]. A common surgical procedure used by maxillofacial surgeons is genioplasty [5]. There are different types such as advancement, rotational, setback, alloplastic, and reduction genioplasty [6]. Genioplasty can be done alone or in conjunction with other osteotomies to attain better chin symmetry [7]. Most of the changes in the chin are achieved in a single-step surgery.

Hofer performed sliding genioplasty for the first time to advance the receding chin by using an extraoral approach [5]. Converse published his work on chin augmentation using bone graft through an intraoral approach [8]. Gillies and Kristensen used the bovine cartilage, while Newman reported the use of dermis graft for chin augmentation [9]. In 1957, Trauner and Obwegeser introduced the intraoral approach for sliding osteotomy of the chin [10]. Correction of the “witch's chin” deformity with an external triangular skin excision was first described by Gonzales-Ulloa in 1972 [11]. In 1978, Loeb described the usage of submental fat flap to improve the unaesthetic shape of the chin [12].

In the past few years, the development of 3D technology has improved the accuracy and outcomes of chin surgeries. For diagnosis and treatment planning, computed tomography (CBCT) and wrapped CBCT images of nonstandardized facial photographs are accurate and can be used to analyze soft tissue profile measurements [13]. However, consideration should be given to minimum radiation or radiation-free diagnostic exam and follow-up [14]. Surgical templates can be designed according to the preoperative virtual surgical plan (VSP) which helps in guiding the accurate osteotomy in maxillofacial surgeries [15]. Total face approach (TFA), a novel 3D method to describe the main cephalometric parameters, can be considered in planning of orthognathic surgery and ancillary surgeries such as reduction and augmentation genioplasty [16]. These latest advancements help to achieve accurate, safe, and predictable single-stage surgery and avoid many preoperative and postoperative complications [17].

In the literature, different surgical techniques and methodologies have been explained, but the biological implications, economic factors, and available resources influence the surgeon's decision in the selection of the best possible treatment protocol [5]. However, genioplasty has a steep and long learning curve, and the complication rates range from 3% to 30% with the average of 10% by a plastic surgeon survey [17]. Most common complications are sensory deficits in the chin (6.46%) followed by infection (5.95%) [18].

The objective of our study is to focus on different types of genioplasties, the technique used, and the incidence of associated postoperative complications. Several studies on complications after mandible surgery have been published, but most of these are based on bilateral sagittal split ramus osteotomy or vertical ramus osteotomy. Furthermore, the surgeries were performed by multiple plastic surgeons at different clinics. There are scarce data in the maxillofacial literature regarding complications specific to genioplasty done by a single surgeon. This study will help postgraduate residents and young surgeons to understand the complications that are common after genioplasty and how to avoid them.

## 2. Materials and Methods

We prospectively reviewed the patients who attended for facial asymmetry at Khyber College of Dentistry, Peshawar, over a six-year period. All patients were included consecutively. Patient's biographic data, type of genioplasty, concomitant orthognathic surgery (if performed), age, and postoperative complications were recorded.

All the patients who had undergone genioplasty primarily for the improvement of facial aesthetics were included in the study. However, patients who were born with congenital deformities were excluded from the study. 59 patients, 21 women and 38 men, age range: 16–55 years, with varying degrees of chin abnormalities were enrolled in this study from March 2015 to April 2020.

The patients were divided into four groups on the basis of the surgery performed:

1. Chin advancement
2. Rotations and advancements
3. Setback
4. Reduction

It was also recorded whether concomitant orthognathic surgery was performed or not. The postoperative complications were also divided into 5 common types: paresthesia of the mental nerve, infection, hematoma, delayed healing of the incision, and bad splits.

The procedure was performed under general anesthesia via orotracheal or nasotracheal intubation. Intraoral approach was used, and labial incision extending from canine to canine was given. The mentalis muscle was identified, and subperiosteal dissection was done while taking care of the mental nerve. Prior to osteotomy, bone marks were made using bur. Along the markings, osteotomy was done using an oscillating saw. The division is completed by a twist of the osteotome. The bony fragment was advanced, rotated, or reduced in the calculated amount as per treatment planning. Fixation was done using screws and titanium miniplates after bending of plates. Bony irregularities were smoothened, copious irrigation was done, and hemostasis was endured. Repair of the mentalis muscle followed by repair of mucosa was done. Light dressing was applied to the submental region to prevent swelling and necrosis of skin. Antibiotics and painkillers were prescribed. Patients were discharged one day after the surgery in case of uneventful recovery. All the surgeries were performed by the first author. All 59 patients underwent CT scan 1 week postoperatively, and all patients had at least 1-month, 3-month, and six-month follow-up, which allowed to evaluate the surgical outcomes.

Approval to carry out the study was sought from the Institutional Ethical Review Committee at Khyber College of Dentistry. Informed consent was obtained from all the patients.The collected data were analyzed using Statistical Package for Social Sciences (SPSS) version 23. Patients and operational characteristics were analyzed using descriptive statistics. Categorical variables were described using absolute counts and percentages. Continuous variables were described as the mean, median, SD, and range. Mean ± SD was calculated for numerical values such as age. Patients were divided into two age groups: 16–35 and 36–55. The postoperative complications were stratified among gender and type of genioplasty. Poststratification chi square test was applied. A probability of 0.05 was kept as significant.

## 3. Results

A prospective review of all cases of genioplasty operated by the first author over 6 years from March 2014 to April 2020 is included. During this time, 59 patients were operated upon. 38 were males and 21 were females with the age range of 16–55 (mean: 27.3729, Std. deviation: 4.70472).

The most common age group was from 16 to 35 years (50.8%). The patients in age group 36 to 55 years were 49.2%. Details are given in Table 1.

There were three main types of genioplasties performed: horizontal osteotomy with advancement, horizontal osteotomy with AP reduction, and correction of asymmetry by rotation/advancement. In 67.8% of patients, rotation/advancement procedure was performed, in 6.9%, horizontal osteotomy with AP reduction was performed, and in 15.3%, horizontal osteotomy with advancement was performed. Details are given in Table 2.

In 17 (28.8%) patients, genioplasty was performed as an adjunct procedure with orthognathic surgery for the correction of maxillomandibular asymmetries. In 42 (71.2%) cases, genioplasty was performed as an isolated procedure for correction chin deformities. Details are given in Table 3.

Among 59 cases, 2 patients (3.4%) developed infection, which was subsided by oral antibiotics. Five patients (8.5%) had hematoma that resolved spontaneously. Four patients (6.8%) had temporary numbness, one of which lasted for more than twelve months. In 3 cases, neurosensory disturbances were resolved after one month. Postoperative complications were seen more in men than in woman. Details and *P* value are given in Table 4.

The postoperative complications were seen more commonly in males than in females, and this association was statistically significant. Details are given in Table 5.

Postoperative complications are also significantly associated with the type of surgery performed. Correction of chin asymmetry by rotation/advancement showed the highest number of complications (84%). Details and *P* value are given in Table 6.

## 4. Discussion

The expression of the chin is equated with character traits, and thus, it is an important component of the profile forms. Two main therapeutic approaches can be used to address chin deformities, alloplastic implants and basal osteotomy of the chin or genioplasty. The latter is the most widely used because of its great versatility to correct three-dimensional chin deformities through osteotomy angle variation with lower rates of postoperative complications [19]. Genioplasty provides functional and aesthetic improvements, and that is why it is a procedure of choice by many surgeons [20]. Genioplasty is one of the significant surgical procedures, and asymmetry, excess, or deficiency of the chin is mainly corrected. Thus, for any surgeon, it is mandatory to be well versed in the surgical technique of genioplasty, and he should be aware of complications that might occur in this surgery. Understanding of complications and management of those complications are mainstay of any surgical treatment [21].

Owing to a very low complication rate, the genioplasty is considered one of the most successful operational activities in the aesthetic plastic surgery. In a study on 200 patients who underwent genioplasty in isolation or combined with other surgical procedures, Richard et al. described only six complications. Fractures, atypical osteotomies, bleeding, soft tissue damage, or nerve injuries are among possible intraoperative complications. Postoperative complications include sensory loss, hematoma, infection, secondary dislocations, bone necrosis, ptosis of the chin, deficient ossification, dental lesions, periodontal lesions, and irregular contours of the lower jaw [22]. In another study, infections, extrusions, and bone erosions were mentioned as the most common complications of genioplasty [23]. In our study, infection, hematoma, and temporary numbness were the most common complications. Two patients (3.4%) developed infection, which was subsided by oral antibiotics. Five patients (8.5%) had hematoma that resolved spontaneously. Four patients had temporary numbness, one of which lasted for more than twelve months. In 3 cases, neurosensory disturbances were resolved after one month.

Neurosensory disturbances reduce the patient satisfaction level by great percentage [24]. The literature reports that if genioplasty is done alone, the incidence of neurosensory injury is low. However, if genioplasty is done concomitantly with orthognathic surgery, the chances of numbness postoperatively are higher [25]. Lindquist and Obeid showed in a study with 31 patients that only 10% of those who underwent isolated genioplasty had nerve alteration, whereas the incidence was of 28.5% in those who underwent genioplasty in combination with sagittal split osteotomy of the mandibular ramus [26]. Our study showed similar results. The incidence of complications was more in patients with concomitant orthognathic surgery. So, while performing orthognathic surgery with genioplasty, careful surgical planning, marking of the incision, and good surgical technique shall be performed to avoid degloving of the chin and nerve damage.

## 5. Conclusion

Temporary neurosensory disturbance of the inferior alveolar nerve is the most common complication that occurs after genioplasty. Patients shall be counselled preoperatively, and informed consent shall be obtained prior to surgery. Careful preoperative planning, marking of the incision in soft tissue and bone markings with drills, minimal retraction of the nerve, and light dressing of the submental region can help the prevention of complications.

## Figures and Tables

**Table 1:** Age group of patients.

Age groups	Frequency	Percent
16–35	30	50.8
36–55	29	49.2
Total	55	100.0

**Table 2:** Type of genioplasty.

Genioplasty	Frequency	Percent
Horizontal osteotomy with advancement	9	15.3
Horizontal osteotomy with AP reduction	10	16.9
Correction of asymmetry by rotation/advancement	40	67.8
Total	55	100.0

**Table 3:** Concomitant mandibular osteotomies.

Concomitant osteotomies	Frequency	Percent
Yes	17	28.8
No	42	71.2
Total	59	100.0

**Table 4:** Postoperative complications.

Postoperative complications	Frequency	Percent
Neurosensory disturbances	4	6.8
Infection	2	3.4
Hematoma	5	8.5
None	48	81.4
Total	59	100

**Table 5:** Postoperative complications stratified by gender.

Gender	Postoperative complications				*P* value
	Neurosensory disturbances	Infection	Hematoma	None	
Male	17	1	3	17	
Female	8	1	2	10	0.0004
Total	25	2	5	27	

**Table 6:** Postoperative complications stratified by type of genioplasty.

Genioplasty type	Postoperative complications								Total		*P* value
	Neurosensory disturbances		Infection		Hematoma		None				
	%	*N*	%	*N*	%	*N*	%	*N*	%	*N*	
Horizontal osteotomy with advancement	0	0	50	1	40	2	22	6	15.3	9	
Horizontal osteotomy with AP reduction	16	4	0	0	0	0	22.2	6	16.9	10	<0.001
Correction of asymmetry by rotation/advancement	84	21	50	1	60	3	55.6	15	67.8	40	
Total	100	25	100	2	100	5	100	27	100	55	

## Data Availability

The data used to support the findings of this study are available from the corresponding author (nigamsattarkhan@gmail.com) upon request.

## References

[1] Niechajev I. (2020). Reduction genioplasty for mandibular prognathism and long chin. *Oral and Maxillofacial Surgery*.

[2] Drissi Qeytoni H., Zribi A., Raphaël B., Lebeau J., Bettega G. (2007). Les génioplasties: techniques et applications. *Revue de Stomatologie et de Chirurgie Maxillo-Faciale*.

[3] Khalifa G. A., Mohamed F. I. (2018). Aesthetic outcomes and morphological changes in chin parameters after mandibular distraction and subsequent advancement genioplasty in patients with mandibular micrognathia. *International Journal of Oral and Maxillofacial Surgery*.

[4] Bertossi D., Albanese M., Mortellaro C. (2018). Osteotomy in genioplasty by piezosurgery. *Journal of Craniofacial Surgery*.

[5] Bertossi D., Galzignato P.-F., Albanese M., Botti C., Botti G., Nocini P. F. (2015). Chin microgenia: a clinical comparative study. *Aesthetic Plastic Surgery*.

[6] Stanton D. C. (2003). Genioplasty. *Facial Plastic Surgery*.

[7] Nadjmi N., Van Roy S., Van de Casteele E. (2017). Minimally invasive genioplasty procedure. *Plastic and Reconstructive Surgery - Global Open*.

[8] Converse J. M. (1946). Restoration of facial contour by bone grafts introduced through the oral cavity. *Plastic and Reconstructive Surgery*.

[9] Gillies S. H., Kristensen H. K. (1951). Ox cartilage in plastic surgery. *British Journal of Plastic Surgery*.

[10] Trauner R., Obwegeser H. (1957). The surgical correction of mandibular prognathism and retrognathia with consideration of genioplasty. *Oral Surgery, Oral Medicine, Oral Pathology*.

[11] González-Ulloa M. (1972). Ptosis of the chin. *Plastic and Reconstructive Surgery*.

[12] Loeb R. (1978). Surgical elimination of the retracted submental fold during double chin correction. *Aesthetic Plastic Surgery*.

[13] Alhammadi M. S., Al-mashraqi A. A., Alnami R. H. (2021). Accuracy and reproducibility of facial measurements of digital photographs and wrapped cone beam computed Tomography (CBCT) photographs.

[14] Reda R., Zanza A. (2021). An update of the possible applications of magnetic resonance imaging (MRI) in Dentistry: a literature review.

[15] Shen S., Jiang T., Shen S. G., Wang X. (2019). A reversed approach for simultaneous mandibular symphyseal split osteotomy and genioplasty. *International Journal of Oral and Maxillofacial Surgery*.

[16] Perrotti G., Baccaglione G., Clauser T., Testarelli L., Del Fabbro M., Testori T. (2021). Total Face Approach (TFA): a novel 3D approach to describe the main cephalometric craniomaxillofacial parameters. *Methods and Protocols*.

[17] Fu X., Qiao J., Girod S. (2017). Standardized protocol for virtual surgical plan and 3-dimensional surgical template-assisted single-stage mandible contour surgery. *Annals of Plastic Surgery*.

[18] Kang M. (2014). Incidence of complications associated with mandibuloplasty. *Plastic and Reconstructive Surgery Global Open*.

[19] Avelar R. L., Sá C. D. L., Esses D. F. S., Becker O. E., Soares E. C. S., de Oliveira R. B. (2014). Unusual complication after genioplasty. *Journal of Craniofacial Surgery*.

[20] Dennis T., Bains A., Doumpiotis D. (2019). Correction of a genioplasty. *British Journal of Oral and Maxillofacial Surgery*.

[21] Ferretti C., Reyneke J. P. (2016). Genioplasty. *Atlas of the Oral and Maxillofacial Surgery Clinics*.

[22] Abadi M., Pour O. B. (2015). Genioplasty. *Facial Plastic Surgery*.

[23] Baus A., Rem K., Revol M., Cristofari S. (2018). Prosthetic genioplasty versus osseous genioplasty in aesthetic chin augmentation: literature review and knowledge update. *Annales de Chirurgie Plastique Esthetique*.

[24] He P., Iwanaga J., Matsushita Y. (2017). A comparative review of mandibular Orthognathic surgeries with a focus on intraoral vertico-sagittal ramus Osteotomy. *Cureus*.

[25] Westermark A., Bystedt H., von Konow L. (1998). Inferior alveolar nerve function after mandibular osteotomies. *British Journal of Oral and Maxillofacial Surgery*.

[26] Lindquist C. C., Obeid G. (1988). Complications of genioplasty done alone or in combination with sagittal split-ramus osteotomy. *Oral Surgery, Oral Medicine, Oral Pathology*.

